# Only 10% of Patients With a Concomitant MCL Injury Return to Their Preinjury Level of Sport 1 Year After ACL Reconstruction: A Matched Comparison With Isolated ACL Reconstruction

**DOI:** 10.1177/19417381231157746

**Published:** 2023-03-10

**Authors:** Eleonor Svantesson, Ramana Piussi, Susanne Beischer, Christoffer Thomeé, Kristian Samuelsson, Jón Karlsson, Roland Thomeé, Eric Hamrin Senorski

**Affiliations:** Department of Orthopaedics, Institute of Clinical Sciences, Sahlgrenska Academy, University of Gothenburg, Gothenburg, Sweden, Sahlgrenska Sports Medicine Center (SSMC), Gothenburg, Sweden; Sahlgrenska Sports Medicine Center (SSMC), Gothenburg, Sweden, Sportrehab, Sport Medicine Clinic, Gothenburg, Sweden, Department of Health and Rehabilitation, Institute of Neuroscience and Physiology, Sahlgrenska Academy, University of Gothenburg, Gothenburg, Sweden; Sportrehab, Sport Medicine Clinic, Gothenburg, Sweden, Department of Health and Rehabilitation, Institute of Neuroscience and Physiology, Sahlgrenska Academy, University of Gothenburg, Gothenburg, Sweden; Sportrehab, Sport Medicine Clinic, Gothenburg, Sweden; Department of Orthopaedics, Institute of Clinical Sciences, Sahlgrenska Academy, University of Gothenburg, Gothenburg, Sweden, Sahlgrenska Sports Medicine Center (SSMC), Gothenburg, Sweden, Department of Orthopedics, Sahlgrenska University Hospital, Mölndal, Sweden; Department of Orthopaedics, Institute of Clinical Sciences, Sahlgrenska Academy, University of Gothenburg, Gothenburg, Sweden, Sahlgrenska Sports Medicine Center (SSMC), Gothenburg, Sweden, Department of Orthopedics, Sahlgrenska University Hospital, Mölndal, Sweden; Sportrehab, Sport Medicine Clinic, Gothenburg, Sweden, Department of Health and Rehabilitation, Institute of Neuroscience and Physiology, Sahlgrenska Academy, University of Gothenburg, Gothenburg, Sweden; Sahlgrenska Sports Medicine Center (SSMC), Gothenburg, Sweden, Sportrehab, Sport Medicine Clinic, Gothenburg, Sweden, Department of Health and Rehabilitation, Institute of Neuroscience and Physiology, Sahlgrenska Academy, University of Gothenburg, Gothenburg, Sweden

**Keywords:** anterior cruciate ligament, medial collateral ligament, reconstruction, return to sport

## Abstract

**Background:**

There is a need for an increased understanding of the way a concomitant medial collateral ligament (MCL) injury may influence outcome after anterior cruciate ligament (ACL) reconstruction.

**Hypothesis:**

Patients with a concomitant MCL injury would have inferior clinical outcomes compared with a matched cohort of patients undergoing ACL reconstruction without an MCL injury.

**Study Design:**

Matched registry-based cohort study; case-control.

**Level of Evidence:**

Level 3.

**Methods:**

Data from the Swedish National Knee Ligament Registry and a local rehabilitation outcome registry were utilized. Patients who had undergone a primary ACL reconstruction with a concomitant nonsurgically treated MCL injury (ACL + MCL group) were matched with patients who had undergone an ACL reconstruction without an MCL injury (ACL group), in a 1:3 ratio. The primary outcome was return to knee-strenuous sport, defined as a Tegner activity scale ≥6, at the 1-year follow-up. In addition, return to preinjury level of sport, muscle function tests, and patient-reported outcomes (PROs) were compared between the groups.

**Results:**

The ACL + MCL group comprised 30 patients, matched with 90 patients in the ACL group. At the 1-year follow-up, 14 patients (46.7%) in the ACL + MCL group had return to sport (RTS) compared with 44 patients (48.9%) in the ACL group (*P* = 0.37). A significantly lower proportion of patients in the ACL + MCL group had returned to their preinjury level of sport compared with the ACL group (10.0% compared with 25.6%, adjusted *P* = 0.01). No differences were found between the groups across a battery of strength and hop tests or in any of the assessed PROs. The ACL + MCL group reported a mean 1-year ACL-RSI after injury of 59.4 (SD 21.6), whereas the ACL group reported 57.9 (SD 19.4), *P* = 0.60.

**Conclusion:**

Patients with a concomitant nonsurgically treated MCL injury did not return to their preinjury level of sport to the same extent as patients without an MCL injury 1 year after ACL reconstruction. However, there was no difference between the groups in terms of return to knee strenuous activity, muscle function, or PROs.

**Clinical Relevance:**

Patients with a concomitant nonsurgically treated MCL injury may reach outcomes similar to those of patients without an MCL injury 1 year after an ACL reconstruction. However, few patients return to their preinjury level of sport at 1 year.

Although a medial collateral ligament (MCL) injury is the most common knee ligament injury,^4,23,37^ research related to the treatment of patients with an MCL injury has historically not attracted a great deal of attention in the literature. Some authors have even referred to it as the “neglected ligament.”^41^ Recently, however, an increased understanding of the association between the failure of anterior cruciate ligament (ACL) reconstruction and a concomitant nonsurgically treated MCL injury^3,32^ has led to a greater appreciation of and interest in the stabilizing properties of the MCL.^10^ The synergy between the ACL and MCL for knee joint stability implies that, when an MCL deficiency is present, the ACL is subjected to increased loads.^11,42^ It has been suggested that supraphysiological loads on an ACL graft after ACL reconstruction increase the risk of graft failure, and several recent studies support this theory by indicating that there is an increased risk of ACL reconstruction failure when a concomitant MCL injury is present.^2,3,22,32^

The way functional performance and patient-reported outcome (PRO) differ between patients undergoing an ACL reconstruction for an isolated ACL injury and those undergoing an ACL reconstruction with a concomitant nonsurgically treated MCL injury is, however, less well understood. A previous study reported that the absence of a concomitant MCL injury was associated with a 7-fold increase in the odds of a return to sport (RTS) within the first year after the ACL reconstruction.^19^ However, this analysis was based on a limited number of patients with an MCL injury and no account was taken of the treatment of the actual MCL injury.^19^ The question of how patients with a concomitant nonsurgically treated MCL injury perform in comparison with those undergoing an ACL reconstruction after an isolated ACL injury in terms of muscle strength, PROs and RTS therefore remains. This could be important knowledge, because if differences exist in the postoperative rehabilitation profile between the groups, this may further explain the increased risk of ACL failure in patients with a concomitant nonsurgically treated MCL injury, as well as revealing modifiable factors that may be used to tailor the rehabilitation protocol more specifically depending on the injury profile.

The purpose of this study was to evaluate whether outcome, in terms of muscle function and PROs, including RTS, differs between patients undergoing an ACL reconstruction with a concomitant nonsurgically treated MCL injury and a matched cohort of patients undergoing an ACL reconstruction for an isolated ACL injury. In the light of recent studies, reporting an increased risk of revision ACL reconstruction in patients with a concomitant MCL injury,^2,3,22,32^ the hypothesis was that patients with a concomitant nonsurgically treated MCL injury would have inferior muscle function, report inferior PROs, and have a lower RTS rate compared with patients with an isolated ACL injury.

## Methods

This study was based on prospectively collected data from 2 registries: the Swedish National Knee Ligament Registry (SNKLR) and Project ACL. Ethical approval for this study was obtained from the Swedish Ethical Review Authority (EPM number: 2020-02501). Participation in Project ACL is voluntary for all parties (patients, physical therapists, and surgeons). In the same way, participation for patients and surgeons in the SNKLR is voluntary. All parties are able to withdraw from participation at any time, without any further explanation.

The SNKLR was established in 2005 and covers over 90% of all ACL reconstructions performed annually in Sweden.^14^ Information about the surgical details for each patient is entered via a standardized form by the surgeon on the date of surgery and it includes, but is not limited to, a specification of the ACL reconstruction, concomitant injuries and procedures, as well as the activity performed at the time the ACL injury was sustained. In the event of any reoperations or revision surgeries, these are entered as separate entities and linked to the patient’s index operation. The patient-reported section of the registry includes validated questionnaires that are administered preoperatively and at 1, 2, 5, and 10 years postoperatively thereafter. Details of the data collection have been given previously.^1,20^ In this study, the data from the SNKLR were used to identify information about the ACL reconstruction, concomitant injuries, demographic data and the 1-year Knee injury and Osteoarthritis Outcome Score (KOOS).^31^

Project ACL is a rehabilitation outcome registry based in the southwestern part of Sweden and was established in September 2014.^18^ A web-based protocol is used regularly to follow up on patients with an ACL injury. The registry comprises various validated PROs, as well as validated lower extremity muscle strength tests and hop tests. Evaluations are scheduled at predefined time points of follow-up (10 weeks, 4, 8, 12, 18, and 24 months, and then yearly up to 5 years), starting with the ACL injury or reconstruction as the baseline. The test includes a battery of hop tests^17^ and knee extension and flexion strength,^27^ as well as PROs. In this study, Project ACL was used to retrieve data on strength and hop tests, as well as PROs.

### Eligibility Criteria

Patients registered in Project ACL with at least a 1-year follow-up after the primary ACL reconstruction were assessed for eligibility, and complementary data on surgical details, concomitant injuries, and the KOOS were extracted from the SNKLR database. The inclusion criteria were a primary ACL reconstruction with a concomitant nonsurgically treated MCL injury, age ≥15 years at the time of ACL reconstruction, and available data on the preinjury Tegner activity scale (Tegner)^33^ and at the 1-year follow-up. Patients who fulfilled 1 of the following criteria were excluded: age under 15 years at ACL reconstruction, no available 1-year follow-up data, preinjury Tegner <6, contralateral ACL graft harvest, graft types other than hamstring tendon or patellar tendon autografts, double-bundle ACL reconstruction, surgical treatment of MCL injury, concomitant cartilage injury of the International Cartilage Repair Society grades 3 to 4,^13^ concomitant other ligament injury, concomitant fracture, nerve or blood vessel injury, and a new ACL injury within 1 year of the primary ACL reconstruction. The inclusion and exclusion criteria were the same for the matched control group, except that these patients were required to have undergone a primary ACL reconstruction without the presence of a concomitant MCL injury.

### Matching Criteria

Patients with and without a concomitant nonsurgically treated MCL injury were ID matched (1:3), in which sex and the presence of concomitant meniscal injury had to be equal. Age difference was minimized, with a maximum difference of 5 years, and the preinjury Tegner difference was minimized, with a maximum difference of 1 unit. A caliper matching procedure was used to ID match patients with and without a concomitant nonsurgically treated MCL injury in a 1:3 ratio. As a result, 1 patient with an ACL reconstruction and a concomitant nonsurgically treated MCL injury (ACL + MCL injury group) was matched with 3 patients undergoing ACL reconstruction for an isolated ACL injury (ACL group).

### Outcome Measurements

The follow-up for all the outcome assessments in this study took place 1 year after ACL reconstruction. The primary outcome was a return to knee-strenuous sport, defined as a Tegner ≥6^18^ and referred to in this study as RTS. The Tegner ranges from 0 to 10, where a score of 0 represents sick leave or disability because of knee problems, and 10 represents the most knee-strenuous activities.^33^ In addition, a return to the preinjury level of sport was determined, defined as the same or a higher Tegner at the 1-year follow-up compared with the preinjury Tegner.

Secondary outcomes included both PROs and tests of muscle function. In terms of PROs, the KOOS,^30,31^ including the KOOS_4_,^15^ were assessed. The KOOS_4_ is a nonvalidated modified KOOS score, in which all the subscales are summarized to produce 1 score and the subscale of Activities of Daily Living (ADL) is excluded to avoid a ceiling effect.^15^ Moreover, the KOOS patient-acceptable symptom state (PASS) was analyzed.^26^ The KOOS threshold values for an acceptable symptom state have been determined by Muller et al^26^ by asking patients who had undergone ACL reconstruction the following question: “Taking into account all the activity you have during your daily life, your level of pain, and also your activity limitations and participation restrictions, do you consider the current state of your knee satisfactory?.” The cut-off value for each subscale was determined as follows: pain ≥88.9, symptoms ≥57.1, ADL = 100, sport and recreation ≥75, and quality of life ≥62.5.^26^ Finally, the shorter version of the Knee Self-Efficacy Scale (K-SES_18_)^12^ and the ACL-Return to Sport after injury (ACL-RSI)^38^ were assessed. In terms of the K-SES_18_,^12^ this is a modification of the original K-SES questionnaire^34^ and includes 18 items, which are related to the patients’ present and future knee-related self-efficacy in knee-demanding tasks. Each item is scored on a 0 to 10 Likert scale.^12^ The ACL-RSI measures the psychological effect of RTS after ACL reconstruction and consists of 12 items that are ranked on a Likert scale from 0 to 10.^38^ The higher the score, the more positive the psychological response. The final ACL-RSI score is transformed into a mean value on a 0 to 100 scale by adding up the total score of all items, multiplying by 100 and then dividing this score by 120 (total score × 100 of 120).

The strength tests were performed according to a standardized protocol.^27^ Initially, the muscle-strength assessment was performed by applying isometric tests for knee extension and flexion (David F200 DMS-EVE and David F300 DMS-EVE, David Health Solutions Ltd, 2013, Finland). The isometric test was, however, replaced in 2015, when isokinetic concentric tests were instead applied using the Biodex System 4 (Biodex Medical Systems, Shirley, New York). In this study, 1 patient in the ACL + MCL group and 8 patients in the ACL group had undergone isometric testing, which was not considered comparable to the isokinetic concentric tests. As a result, the isometric testing data were excluded in the analysis for muscle testing for these patients and handled as missing data. The following muscle function tests were assessed: unilateral knee extension, knee flexion, vertical hop, hop for distance, and side hop. The testing procedure started with a standardized warm-up procedure of 10 minutes on a stationary bike and submaximum trials on each test. The injured leg was tested first, followed by the uninjured leg. The isokinetic testing was performed at an angular velocity of 90 deg/s, with the patients in a seated position. Three maximum repetitions (with 40 seconds of rest between each repetition) were performed. The highest peak torque was used for analysis. The battery of hop tests included the single-leg vertical hop (jump as high as possible) (Musclelab, Ergotest Innovation AS, Oslo, Norway), the single-leg hop for distance (jump as far as possible), and the single-leg side hop (jump as many times as possible over 2 lines, 40 cm apart, during 30 seconds). For the vertical hop and the hop for distance, 2 practice and 3 test trials were performed. Distance and height were measured in centimeters and the best results in each test were used for analysis. For the side-hop test, one 30-second trial per leg was allowed and the total number of hops was recorded.^28^ Table 1 summarizes the tests of muscle function. The safety restrictions defined by the test protocol, define criteria for situations when the tests cannot be performed, such as if the patient has pain or swelling of the knee at the time of testing. All the functional performance tests were reported as the Limb Symmetry Index (LSI), defined as the outcome for the injured leg divided by the outcome for the uninjured leg multiplied by 100 to obtain the percentage of performance for the injured leg in comparison with the uninjured leg.^35^

**Table 1.** Tests of muscle function^28^

	Degrees of Movement	Practice Trials n (% of 1RM)	Test Trials (n)	Rest between Test Trials (seconds)	Units
Knee extension	90° to 0°	10 (50%); 10 (75%); 1-2 (90%)	3 to 4	40	Newton meters
Knee flexion	0° to 90°	10 (50%); 10 (75%); 1-2 (90%)	3 to 4	40	Newton meters
Vertical hop	-	2	3	20	Centimeters
Hop for distance	-	2	3 to 5	20	Centimeters
Side hop	-	-	1	180	Number of hops

n, number; 1RM, 1 repetition maximum.

### Statistical Analysis

The statistical analyses were performed using Statistical Analysis System software (SAS/STAT, version 14.2, 2016; SAS Institute Inc., Cary, North Carolina). The sample size calculation was made for the main outcome (Tegner ≥6 at the 1-year follow-up), in which the estimated proportion of patients reaching the outcome was based on data in a previous study.^19^ Estimating that 60% of patients with an isolated ACL injury and 30% of patients with an associated MCL injury would report a Tegner ≥6 1 year after ACL reconstruction, 28 patients were required to be included in the MCL group to be able to identify a 30% difference between groups with 80% power at an alpha level of 0.05.

For continuous variables, the mean and SD, as well as the median with range and first and third quartiles, were calculated. Frequency and proportion were calculated for categorical variables. Fisher’s exact test (lowest 1-sided *P* value multiplied by 2) was used to compare dichotomous variables between groups, while the Mantel-Haenszel chi square exact test was used for ordered categorical variables. For continuous variables, Fisher’s nonparametric permutation test was used. The CI for dichotomous variables represented the unconditional exact confidence limits. If no exact limits could be computed, the asymptotic Wald confidence limits with continuity correction were calculated instead. The CI for the mean difference between groups was based on Fisher’s nonparametric permutation test. Because time from injury to ACL reconstruction was found to differ significantly between the groups, the analyses of the 1-year Tegner were adjusted for time from injury to ACL reconstruction by applying logistic regression. All the tests were two-tailed and conducted at the 5% significance level.

## Results

**Figure 1.**
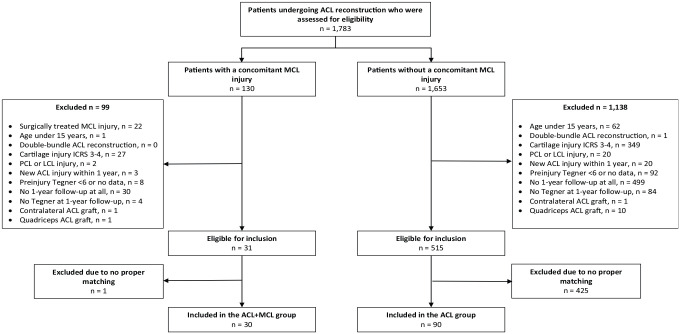
Flowchart of the inclusion process. ACL, anterior cruciate ligament; ICRS, International Cartilage Repair Society; LCL, lateral collateral ligament; MCL, medial collateral ligament; PCL, posterior cruciate ligament.

A total of 30 patients (33.3% women, n = 10) who had undergone a primary ACL reconstruction with a concomitant nonsurgically treated MCL injury met the inclusion criteria and were thus matched with 90 patients who had undergone an ACL reconstruction in the absence of an MCL injury. All the ACL reconstructions took place between January 2014 and October 2019 (see Figure 1).

The mean age at ACL reconstruction was 27.1 (SD 9.6) years in the ACL + MCL group and 26.3 (SD 9.4) years in the ACL group, respectively (*P* = 0.71). The mean time from injury to ACL reconstruction was shorter for the ACL + MCL group compared with the ACL group (mean 114.6 days [SD 78.8] compared with a mean of 384.4 days [SD 928.0], *P* < 0.01). Except for this difference, the groups did not differ significantly in the baseline demographic data for any other variable (Table 2). For the total study population, the most common activity at the time of sustaining the ACL injury was playing soccer (n = 52, 43.3%), followed by alpine skiing (n = 28, 23.3%) (Table 3).

**Table 2.** Demographic data for the study population

	Total (n = 120)	ACL + MCL (n = 30)	ACL (n = 90)	*P* value	Difference between Groups Mean (95% CI)
Patient sex					
Women	40 (33.3 %)	10 (33.3 %)	30 (33.3 %)	1.00	0.0 (-21.7; 21.7)
Age at ACL reconstruction (years)	26.5 (9.4) 23.7 (15.1; 49.6) (18.7; 31.1) n = 120	27.1 (9.6) 23.2 (16; 49.6) (19.2; 33.2) n = 30	26.3 (9.4) 24.2 (15.1; 48.4) (18.6; 30.6) n = 90	0.71	0.807 (-3.298; 4.673)
Time from injury to ACL reconstruction (days)	317.0 (812.0) 133.5 (30; 7451) (85.5; 264.5) n = 120	114.6 (78.8) 92 (33; 357) (56; 158) n = 30	384.4 (928.0) 154 (30; 7451) (97; 321) n = 90	< 0.01	-269.8 (-658.2; -58.5)
ACL graft choice					
Patellar tendon	18 (15.1%)	8 (26.7%)	10 (11.2%)		15.4 (-3.9; 34.8)
Hamstring tendon	101 (84.9%)	22 (73.3%)	79 (88.8%)	0.07	-15.4 (-34.8; 3.9)
Medial meniscal injury					
Yes	21 (17.5%)	4 (13.3%)	17 (18.9%)	0.70	-5.6 (-22.4; 11.3)
Lateral meniscal injury					
Yes	47 (39.2%)	14 (46.7%)	33 (36.7%)	0.45	10.0 (-12.7; 32.7)
Cartilage injury					
Yes	9 (7.5%)	2 (6.7%)	7 (7.8%)	1.00	-1.1 (-13.8; 11.6)
Preinjury Tegner activity scale					
6	20 (16.7%)	6 (20.0%)	14 (15.6%)		
7	26 (21.7%)	6 (20.0%)	20 (22.2%)		
8	25 (20.8%)	5 (16.7%)	20 (22.2%)		
9	35 (29.2%)	7 (23.3%)	28 (31.1%)		
10	14 (11.7%)	6 (20.0%)	8 (8.9%)	1.00	
Preoperative KOOS					
Symptoms	72.7 (16.7) 75 (32.1; 100) (60.7; 85.7) n = 99	69.3 (15.8) 67.9 (32.1; 100) (60.7; 78.6) n = 27	74.0 (17.0) 78.6 (35.7; 100) (60.7; 89.3) n = 72	0.23	-4.70 (-12.14; 3.01)
Pain	76.1 (15.6) 77.8 (38.9; 100) (69.4; 88.9) n = 99	77.1 (15.3) 77.8 (38.9; 100) (69.4; 88.9) n = 27	75.7 (15.9) 79.2 (41.7; 100) (68.1; 88.9) n = 72	0.72	1.32 (-5.56; 8.50)
Activities of Daily Living	85.1 (15.0) 89.7 (47.1; 100) (76.5; 97.1) n = 99	83.7 (15.9) 89.7 (50; 100) (76.5; 97.1) n = 27	85.6 (14.7) 92.6 (47.1; 100) (75.7; 97.1) n = 72	0.56	-1.96 (-8.60; 5.02)
Sports and recreation	39.8 (24.4) 45 (0; 100) (20; 55) n=99	34.8 (24.4) 35 (0; 100) (15; 50) n=27	41.7 (24.3) 45 (0; 85) (20; 57.5) n=72	0.23	-6.85 (-17.94; 4.21)
Quality of life	34.0 (16.1) 31.3 (0; 87.5) (25; 43.8) n = 99	36.6 (18.6) 31.3 (6.3; 87.5) (25; 50) n = 27	33.0 (15.0) 34.4 (0; 68.8) (18.8; 43.8) n = 72	0.35	3.59 (-3.82; 10.76)
KOOS_4_	55.6 (15.1) 57.9 (21.4; 96) (44.5; 65.2) n = 99	54.4 (14.9) 56.1 (21.4; 96) (44.5; 63.8) n = 27	56.1 (15.2) 59.3 (24.6; 85.5) (44.8; 68.4) n = 72	0.64	-1.66 (-8.53; 5.16)

ACL, anterior cruciate ligament; KOOS, Knee injury and Osteoarthritis Outcome Score; MCL, medial collateral ligament.

For categorical variables, n (%) is presented.

For continuous variables, the mean (SD)/median (min; max)/ Q1; Q3)/n = is presented. The mean difference between groups is calculated as the MCL + ACL group minus the ACL group.

**Table 3.** Physical activity at the time of anterior cruciate ligament injury for the study population

	Total (n = 120)	ACL + MCL (n = 30)	ACL (n = 90)
Activity at the time of ACL injury			
Soccer	52 (43.3%)	13 (43.3%)	39 (43.3%)
Alpine skiing	28 (23.3%)	9 (30.0%)	19 (21.1%)
Handball	16 (13.3%)	2 (6.7%)	14 (15.6%)
Floorball	6 (5.0%)	2 (6.7%)	4 (4.4%)
Others	18 (15.0%)	4 (13.3%)	14 (15.6%)

ACL, anterior cruciate ligament; MCL, medial collateral ligament.

For categorical variables, n (%) is presented.

### Return to Sport

At the 1-year follow-up, 14 patients (46.7%) in the ACL + MCL group had an RTS defined as reporting a Tegner ≥6, which did not differ from the ACL group (n = 44, 48.9%). A total of 3 patients (10.0%) in the ACL + MCL group had reached their preinjury level of sport at 1 year, compared with 23 patients (25.6%) in the ACL group, which represented a significantly lower return to preinjury rate for the MCL group when adjusting for time from injury to ACL reconstruction (*P* = 0.01) (Table 4).

**Table 4.** The Tegner activity score at the 1-year follow-up

	Total (n = 120)	ACL + MCL (n = 30)	ACL (n = 90)	*P* value	Adjusted *P* value*	Difference between Groups Mean (95% CI)
Return to sport (Tegner score ≥6)						
Yes	58 (48.3%)	14 (46.7%)	44 (48.9%)	1.00	0.37	-2.2 (-25.1; 20.6)
Return to preinjury Tegner activity level						
Yes	26 (21.7%)	3 (10.0%)	23 (25.6%)	0.11	0.01	-15.6 (-31.8; 0.7)

ACL, anterior cruciate ligament; MCL, medial collateral ligament.

For categorical variables, n (%) is presented. The mean difference between groups is calculated as the MCL + ACL group minus the ACL group.

* Adjusting for time from injury to ACL reconstruction using logistic regression.

### Test of Muscle Function

The mean LSI for hamstring and quadriceps strength did not differ between the groups (Table 5). For quadriceps strength, the mean LSI was 92.8% (SD 10.0%) for the ACL + MCL group and 94.4% (SD 8.5%) for the ACL group (*P* = 0.45). The LSI for hamstring strength was 99.2% (SD 11.9%) for the ACL + MCL group and 98.4% (SD 11.8%) for the ACL group (*P* = 0.76). With regard to the hop tests, there were no differences in the LSI between the groups in any of the 3 hop tests (Table 5). The absolute numbers of the muscle strength tests and the hop tests for each group are presented in Appendix 1 (available in the online version of this article).

**Table 5.** The Limb Symmetry Index for muscle strength and hop tests

	Total (n = 120)	ACL + MCL (n = 30)	ACL (n = 90)	*P* value	Difference between Groups Mean (95% CI)
Quadriceps strength LSI (%)	94.0 (8.9) 95.4 (71.9; 123.5) (87.5; 99.3) n = 92	92.8 (10.0) 94.6 (71.9; 117.3) (86.6; 100) n = 25	94.4 (8.5) 95.6 (72.6; 123.5) (89.3; 99.3) n = 67	0.45	-1.6 (-5.7; 2.6)
Hamstring strength LSI (%)	98.6 (11.8) 97.8 (64.5; 128.6) (90.6; 106.8) n = 92	99.2 (11.9) 100 (64.5; 122.5) (92.9; 108.3) n = 25	98.4 (11.8) 97.3 (72.8; 128.6) (89.5; 105.2) n = 67	0.76	0.8 (-4.7; 6.4)
Single-leg vertical hop LSI (%)	85.7 (15.9) 86.3 (41; 123.4) (76.9; 96.1) n = 91	85.8 (17.4) 89.1 (46.4; 123.4) (72.2; 96.1) n = 23	85.7 (15.6) 86.3 (41; 118) (77; 96) n = 68	0.98	0.2 (-7.7; 7.9)
Single-leg hop for distance LSI (%)	94.0 (8.6) 95 (68.6; 111.2) (89.5; 100.7) n = 91	96.1 (7.3) 93 (80; 111.2) (90.9; 101.2) n = 23	93.3 (9.0) 95.1 (68.6; 111.1) (88.1; 99.7) n = 68	0.17	2.9 (-1.1; 7.0)
Single-leg side hop LSI (%)	94.2 (13.7) 93.6 (50; 136.8) (88.5; 102.4) n = 86	97.4 (12.6) 94.8 (75; 136.8) (89.5; 105.3) n = 23	93.0 (14.0) 93.4 (50; 122.7) (87; 100) n = 63	0.18	4.5 (-2.0; 11.2)

ACL, anterior cruciate ligament; LSI, Limb Symmetry Index; MCL, medial collateral ligament

For continuous variables, the mean (SD)/median (min; max)/(Q1; Q3). The mean difference between groups is calculated as the MCL + ACL group minus the ACL group.

### Patient-Reported Outcome

The 1-year KOOS did not differ between the groups on any subscale. The KOOS_4_ was 76.0 (SD 16.0) for the ACL + MCL group and 75.7 (SD 13.7) for the ACL group (*P* = 0.97). A majority of the patients in both groups reached the KOOS PASS for all subscales except the ADL subscale. The subscale with the highest proportion of achieving a PASS was the symptom subscale for both groups, in which 90.0% and 94.6% reported a score above the threshold for an acceptable symptom state in the ACL + MCL group and the ACL group, respectively (*P* = 0.75). On the sports and recreation subscale, 55.0% of the patients in the ACL + MCL group and 58.1% in the ACL group achieved a PASS (*P* = 1.00).

The K-SES_18_ score did not differ between the groups, in which the ACL + MCL group had a mean K-SES_18_ present of 8.1 (SD 1.7) and the ACL group had a mean of 8.3 (SD 1.4) (*P* = 0.50). The respective scores for the K-SES_18_ future were 7.2 (SD 1.5) and 7.3 (SD 1.9) (*P* = 0.83). Similarly, the ACL-RSI did not differ between the groups. The ACL + MCL group reported a mean 1-year ACL-RSI of 59.4 (SD 21.6), while the ACL group reported a mean score of 57.9 (SD 19.4), (*P* = 0.60). The 1-year PROs are presented in detail in Table 6.

**Table 6.** Comparison of patient-reported outcomes at 1 year postoperatively

	Total (n = 120)	ACL + MCL (n = 30)	ACL (n = 90)	*P* value	Difference between Groups Mean (95% CI)
1-year KOOS					
Symptoms	81.7 (14.1) 85.7 (35.7; 100) (75; 92.9) n = 94	82.7 (15.3) 85.7 (46.4; 100) (80.4; 94.6) n = 20	81.4 (13.8) 85.7 (35.7; 100) (71.4; 92.9) n = 74	0.75	1.26 (-5.58; 8.71)
Pain	88.9 (9.9) 91.7 (61.1; 100) (83.3; 97.2) n = 94	91.7 (7.8) 91.7 (72.2; 100) (86.1; 97.2) n = 20	88.1 (10.3) 91.7 (61.1; 100) (80.6; 97.2) n = 74	0.14	3.57 (-1.11; 8.52)
Activities in Daily Living	96.3 (5.4) 98.5 (70.6; 100) (94.1; 100) n = 94	96.9 (3.8) 97.8 (88.2; 100) (96.3; 100) n = 20	96.2 (5.8) 98.5 (70.6; 100) (94.1; 100) n = 74	0.65	0.727 (-1.765; 3.633)
Sports and recreation	72.2 (21.9) 75 (5; 100) (60; 90) n = 94	68.0 (27.9) 80 (5; 100) (52.5; 92.5) n = 20	73.4 (20.0) 75 (25; 100) (65; 90) n = 74	0.34	-5.38 (-16.15; 6.07)
Quality of life	60.4 (18.7) 62.5 (12.5; 100) (43.8; 75) n = 94	61.6 (19.4) 65.6 (25; 93.8) (46.9; 75) n = 20	60.1 (18.6) 62.5 (12.5; 100) (43.8; 75) n = 74	0.79	1.51 (-7.42; 10.83)
KOOS_4_	75.8 (14.1) 78.4 (40.6; 98.4) (67.3; 86.4) n = 94	76.0 (16.0) 78.4 (40.6; 98.4) (66.4; 87.9) n = 20	75.7 (13.7) 78.6 (43.5; 97.9) (67.8; 85.1) n = 74	0.97	0.240 (-6.652; 7.370)
1-year KOOS PASS					
Symptoms ≥57.1					
Yes	88 (93.6%)	18 (90.0%)	70 (94.6%)	0.75	-4.6 (-21.9; 12.7)
Pain ≥88.9					
Yes	50 (53.2%)	12 (60.0%)	38 (51.4%)	0.67	8.6 (-18.8; 36.1)
Activities of Daily Living = 100					
Yes	42 (44.7%)	8 (40.0%)	34 (45.9%)	0.83	-5.9 (-29.3; 19.7)
Sports and recreation ≥75					
Yes	54 (57.4%)	11 (55.0%)	43 (58.1%)	1.00	-3.1 (-30.8; 24.6)
Quality of Life ≥62.5					
Yes	53 (56.4%)	13 (65.0%)	40 (54.1%)	0.54	10.9 (-16.0; 37.9)
1-year K-SES_18_					
K-SES_**18**_ present	8.3 (1.5) 8.7 (2.9; 10.0) (7.5; 9.4) n = 119	8.1 (1.7) 8.8 (3.4; 10.0) (7.2; 9.5) n = 30	8.3 (1.4) 8.7 (2.9; 10.0) (7.7; 9.4) n = 89	0.50	-0.2 (-0.8; 0.4)
K-SES_18_ future	7.3 (1.8) 7.5 (1.0; 10.0) (6.0; 8.8) n = 119	7.2 (1.5) 7.2 (3.8; 9.5) (6.0; 8.5) n = 30	7.3 (1.9) 8.0 (1.0; 10.0) (6.0; 8.8) n = 89	0.83	-0.1 (-0.8; 0.7)
1-year ACL-RSI	57.6 (19.9) 57.5 (16.7; 100) (42.5; 72.5) n = 107	59.4 (21.6) 55.8 (25; 95) (40.8; 80) n = 27	57.0 (19.4) 57.5 (16.7; 100) (43.3; 70.8) n = 80	0.60	2.42 (-6.49; 11.40)

ACL, anterior cruciate ligament; ACL-RSI, anterior cruciate ligament return to sport after injury; KOOS, Knee injury and Osteoarthritis Outcome Score; K-SES, Knee Self-Efficacy Scale; MCL, medial collateral ligament; PASS, patient-acceptable symptom state.

For categorical variables, n (%) is presented.

For continuous variables, the mean (SD)/median (min; max)/Q1; Q3)/n= is presented. The mean difference between groups is calculated as the MCL + ACL group minus the ACL group.

## Discussion

The main finding of this study was that patients with a concomitant nonsurgically treated MCL injury had a lower rate of return to preinjury level of sports at 1 year compared with patients undergoing ACL reconstruction in the absence of MCL injury. Only 10% of the patients in the ACL + MCL group had reached their preinjury level of sport compared with 26% in the ACL group. There were, however, no differences in terms of RTS (Tegner ≥6), muscle function or PROs 1 year postoperatively between the groups. In fact, patients undergoing ACL reconstruction with a concomitant nonsurgically treated MCL injury achieved symmetric muscle strength and reported PROs in line with those who had undergone an ACL reconstruction in the absence of an MCL injury.

Despite the fact that the return to preinjury level of sport was lower in the MCL group, the study results generally did not support the hypothesis of this study, namely that the ACL + MCL group would show an inferior outcome compared with the ACL group. On the one hand, these study results are positive indicators that a more severe injury pattern with a concomitant MCL injury does not need to delay the time needed to regain muscle strength or influence the patients’ knee-related self-efficacy after ACL reconstruction. On the other hand, with recent reports of an increased risk of revision ACL reconstruction for patients with a concomitant MCL injury,^2,3,22,32^ particularly careful decision-making for RTS might be indicated for this population. Persistent medial-side laxity in the event of MCL injury is 1 factor that could increase the risk of ACL failure due to increased forces on the ACL graft.^11,42^ A reduction in the restraining capacity of the MCL may also increase the risk of valgus collapse when either direct or indirect forces are applied to the knee joint. However, as it appears that neither the achievement of symmetrical muscle function nor patient-perceived knee function is what differentiates the rehabilitation phase of the first postoperative year between patients undergoing ACL reconstruction with and without MCL injury, it is reasonable to assume that other factors contribute to the low proportion of RTS and return to preinjury level of sport rates in this cohort, especially for the MCL group. A previous study utilizing the same registries found that the absence of a concomitant MCL injury increased the odds of RTS sevenfold.^19^ The 10% rate of return to preinjury level of sport for the ACL + MCL group is lower than that previously reported in an ACL-reconstructed population,^7,9^ although a higher rate of return to some kind of sport (81%) and a return to the preinjury level of sport (65%) have been reported at a mean follow-up time of 40 months.^6^ The results of the current study should therefore be interpreted with caution, because a 1-year postoperative evaluation after ACL reconstruction is still early.^5,8^ Moreover, a low rate of RTS could indicate a careful progressive rehabilitation process and, in accordance with recent literature, emphasize that patience relating to RTS is important.^25^ Patients need to understand that the progression to RTS takes time and that, despite good LSIs, functional performance, high self-efficacy and psychological readiness, there is no reason to rush the return to preinjury sport. One should also remember that there might be several factors influencing whether a patient decides to return to preinjury sport, which include contextual factors such as level of prior sport participation, motivation and social factors.^6^ Thus, although this study only included patients with a preinjury Tegner ≥6 (ie, had participated in knee-strenuous sport prior to ACL injury) in order to form a study population of active patients, there might be several underlying factors influencing the outcome of returning to preinjury level of sport that this study failed to capture.

It has previously been established that the rate of subsequent ACL reinjury decreases when achieving an LSI of ≥90% across a battery of tests.^16,21^ Although this study did not evaluate the achievement of an LSI of ≥90% across the battery of tests, it is noteworthy that both groups had a mean LSI of >90% in 4 of 5 of the individual muscle function tests. This indicates a generally symmetric muscle function in both groups. The achievement of an LSI of ≥90% across a battery of tests is an important criterion for decision-making relating to the timing of RTS^24^ and should be evaluated in clinical practice. The LSI should, however, be compared with the precapacity,^40^ because it has been shown that approximately one-third of the patients might not have recovered their preoperative absolute muscle strength, despite achieving an LSI ≥90%.^29^ It should be mentioned that the preinjury capacity of the cohorts in this study was not available. In terms of the psychological indicators for RTS, the ACL-RSI has repeatedly been shown to correlate with RTS and a return to the preinjury physical activity level.^7,36,39^ In a recent study, in which 61% of the cohort had returned to their preinjury level of sport performance at a mean follow-up of 2 years, psychological readiness as determined by the ACL-RSI at the 12-month follow-up was the only significant predictor of achieving a return to the preinjury level of sport.^39^ It has previously also been reported that an ACL-RSI <56 might predict failure to return to the preinjury level of sport.^7^ The fact that both groups in the current study reported an overall ACL-RSI >56 indicates that the prognosis for an RTS is likely promising, but further investigation of the cohort with a longer period of follow-up is warranted.

The strengths of this study include the prospectively collected data and the fact that it was a matched comparative study, which should therefore reduce the risk of confounding factors in the comparative analyses between groups. Somewhat surprisingly, however, there was a large discrepancy in the mean time from injury to ACL reconstruction between the groups. Although the reason for this remains unknown, because the indication for treatment is not kept within the registries, one could speculate that preoperative greater functional deficits in the ACL + MCL group might have influenced the decision making for earlier ACL reconstruction in this group. Owing to the potential of confounding the analysis we chose to adjust the analysis for the primary outcome of Tegner ≥6 as well as the return to preinjury Tegner for the time from injury to ACL reconstruction. In terms of limitations, there was a varying degree of loss of data relating to the secondary outcomes, which might have influenced the analysis. The loss to follow-up for the strength and hop test might have been influenced by the safety restrictions defined by the test protocol. In order to match patients, stringent inclusion and exclusion criteria were applied, meaning that the final population was relatively small and the reproduction of the study with a larger population would be a perspective for future research. This could also increase the understanding of the generalizability of current study results, because the study population comprised a smaller proportion of women and the study setting was limited to 1 center. Another limitation of the study is the fact that no power analyses were made for the secondary outcome measures, which means that there is a risk of type II error for the secondary analyses. In addition, both preoperative and postoperative laxity outcome measurements would have strengthened the analysis. The degree of medial side laxity for the MCL injured group preoperatively should most likely have influenced the clinical decision-making of nonoperative treatment, and follow-up assessment of medial side laxity should have yielded a better understanding of the status of the nonsurgically treated MCL injury at the outcome assessment. However, laxity data are currently not available in the registries. There is, nevertheless, a need for a deeper understanding of the functional performance and the psychological factors during the rehabilitation process specifically for ACL-reconstructed patients with a concomitant MCL injury.

## Conclusion

A significantly lower proportion of patients with a concomitant nonsurgically treated MCL injury had returned to their preinjury level of sport 1 year after ACL reconstruction compared with patients without an MCL injury. However, there was no difference between the groups in terms of return to knee strenuous activity, muscle function, or PROs.

## Supplemental Material

sj-docx-1-sph-10.1177_19417381231157746 – Supplemental material for Only 10% of Patients With a Concomitant MCL Injury Return to Their Preinjury Level of Sport 1 Year After ACL Reconstruction: A Matched Comparison With Isolated ACL Reconstruction Click here for additional data file. Supplemental material, sj-docx-1-sph-10.1177_19417381231157746 for Only 10% of Patients With a Concomitant MCL Injury Return to Their Preinjury Level of Sport 1 Year After ACL Reconstruction: A Matched Comparison With Isolated ACL Reconstruction by Eleonor Svantesson, Ramana Piussi, Susanne Beischer, Christoffer Thomeé, Kristian Samuelsson, Jón Karlsson, Roland Thomeé and Eric Hamrin Senorski in Sports Health: A Multidisciplinary Approach
