# Supplementary material for: Only 10% of Patients With a Concomitant MCL Injury Return to Their Preinjury Level of Sport 1 Year After ACL Reconstruction: A Matched Comparison With Isolated ACL Reconstruction
Source: Sports Health. 2023 Mar 10;16(1):124–35. doi: 10.1177/19417381231157746 (PMC10732101; doi:10.1177/19417381231157746)
Supplement: sj-docx-1-sph-10.1177_19417381231157746 – Supplemental material for Only 10% of Patients With a Concomitant MCL Injury Return to Their Preinjury Level of Sport 1 Year After ACL Reconstruction: A Matched Comparison With Isolated ACL Reconstruction [file sj-docx-1-sph-10.1177_19417381231157746.docx]

| **Appendix 1. Strength and hop measurements at the one-year follow-up** | | | | | |
| --- | --- | --- | --- | --- | --- |
|  | **Total (n=120)** | **ACL + MCL (n=30)** | **ACL (n=90)** | **p-value** | **Difference between groups Mean (95% CI)** |
| **Quadriceps strength** |  |  |  |  |  |
| Absolute strength (injured leg) | 191.0 (47.8) 178.5 (100; 366) (157; 221) n=92 | 197.1 (53.9) 183 (120; 366) (160; 219) n=25 | 188.7 (45.5) 177 (100; 309) (157; 228) n=67 | 0.46 | 8.43 (-14.56; 29.59) |
| Absolute strength (uninjured leg) | 203.4 (46.7) 198.5 (113; 327) (169; 241.5) n=92 | 210.9 (43.7) 200 (149; 312) (180; 246) n=25 | 200.6 (47.8) 198 (113; 327) (167; 239) n=67 | 0.34 | 10.4 (-11.9; 31.3) |
| Absolute strength per kg (injured leg) | 2.62 (0.48) 2.61 (1.58; 4.07) (2.32; 2.96) n=92 | 2.73 (0.55) 2.72 (1.62; 4.07) (2.43; 3.05) n=25 | 2.58 (0.45) 2.55 (1.58; 3.86) (2.26; 2.86) n=67 | 0.20 | 0.146 (-0.083; 0.364) |
| Absolute strength per kg (uninjured leg) | 2.80 (0.46) 2.8 (1.7; 3.86) (2.45; 3.13) n=92 | 2.93 (0.42) 3.04 (2.02; 3.6) (2.66; 3.19) n=25 | 2.75 (0.47) 2.78 (1.7; 3.86) (2.43; 3.06) n=67 | 0.09 | 0.183 (-0.031; 0.396) |
| LSI (%) | 94.0 (8.9) 95.4 (71.9; 123.5) (87.5; 99.3) n=92 | 92.8 (10.0) 94.6 (71.9; 117.3) (86.6; 100) n=25 | 94.4 (8.5) 95.6 (72.6; 123.5) (89.3; 99.3) n=67 | 0.45 | -1.57 (-5.74; 2.59) |
| **Hamstring strength** |  |  |  |  |  |
| Absolute strength (injured leg) | 107.1 (27.9) 107 (59; 188) (87; 125.5) n=92 | 111.0 (30.8) 108 (69; 188) (87; 129) n=25 | 105.6 (26.8) 107 (59; 170) (87; 123) n=67 | 0.40 | 5.47 (-7.71; 18.37) |
| Absolute strength (uninjured leg) | 109.2 (27.4) 105 (46; 177) (90; 127) n=92 | 111.8 (26.7) 107 (70; 177) (91; 127) n=25 | 108.3 (27.7) 103 (46; 164) (88; 127) n=67 | 0.58 | 3.55 (-9.37; 16.11) |
| Absolute strength per kg (injured leg) | 1.47 (0.30) 1.49 (0.88; 2.22) (1.25; 1.67) n=92 | 1.54 (0.34) 1.53 (0.88; 2.22) (1.36; 1.77) n=25 | 1.45 (0.29) 1.48 (0.88; 2.1) (1.24; 1.65) n=67 | 0.18 | 0.095 (-0.042; 0.232) |
| Absolute strength per kg (uninjured leg) | 1.50 (0.28) 1.52 (0.81; 2.13) (1.3; 1.65) n=92 | 1.55 (0.28) 1.54 (1.04; 2.12) (1.37; 1.67) n=25 | 1.48 (0.28) 1.48 (0.81; 2.13) (1.27; 1.63) n=67 | 0.25 | 0.074 (-0.053; 0.203) |
| LSI (%) | 98.6 (11.8) 97.8 (64.5; 128.6) (90.6; 106.8) n=92 | 99.2 (11.9) 100 (64.5; 122.5) (92.9; 108.3) n=25 | 98.4 (11.8) 97.3 (72.8; 128.6) (89.5; 105.2) n=67 | 0.76 | 0.845 (-4.697; 6.421) |
| **Single-leg vertical hop** |  |  |  |  |  |
| Vertical hop absolute (injured leg) | 12.6 (5.2) 12.2 (3.2; 29.1) (9.5; 15.4) n=92 | 12.9 (6.2) 12.3 (3.5; 29.1) (10.1; 14.8) n=23 | 12.5 (4.8) 12 (3.2; 25.1) (9.5; 15.5) n=69 | 0.74 | 0.426 (-2.156; 2.860) |
| Vertical hop absolute (uninjured leg) | 14.3 (5.0) 14 (5.1; 30.4) (11.3; 16.2) n=91 | 14.6 (5.6) 14 (5.1; 30.4) (11.4; 15.6) n=23 | 14.2 (4.8) 13.9 (5.3; 29.6) (11.2; 16.8) n=68 | 0.77 | 0.352 (-2.158; 2.683) |
| Vertical hop absolute per kg (injured leg) | 0.175 (0.071) 0.174 (0.038; 0.323) (0.131; 0.221) n=92 | 0.180 (0.079) 0.188 (0.039; 0.323) (0.14; 0.23) n=23 | 0.174 (0.068) 0.174 (0.038; 0.322) (0.131; 0.22) n=69 | 0.70 | 0.007 (-0.028; 0.040) |
| Vertical hop absolute per kg (uninjured leg) | 0.199 (0.068) 0.194 (0.053; 0.389) (0.163; 0.235) n=91 | 0.204 (0.072) 0.189 (0.071; 0.351) (0.166; 0.258) n=23 | 0.198 (0.067) 0.197 (0.053; 0.389) (0.16; 0.233) n=68 | 0.72 | 0.006 (-0.028; 0.039) |
| LSI (%) | 85.7 (15.9) 86.3 (41; 123.4) (76.9; 96.1) n=91 | 85.8 (17.4) 89.1 (46.4; 123.4) (72.2; 96.1) n=23 | 85.7 (15.6) 86.3 (41; 118) (77; 96) n=68 | 0.98 | 0.156 (-7.709; 7.921) |
| **Single-leg hop for distance** |  |  |  |  |  |
| Hop for distance absolute (injured leg) | 122.4 (28.5) 122.5 (46; 215) (107; 141.5) n=92 | 129.8 (31.9) 130 (68; 215) (116; 144) n=23 | 120.0 (27.1) 121 (46; 168) (106; 139) n=69 | 0.16 | 9.87 (-3.60; 23.94) |
| Hop for distance absolute (uninjured leg) | 129.4 (26.7) 128 (59; 202) (112; 147) n=91 | 134.6 (29.3) 135 (85; 202) (112; 154) n=23 | 127.7 (25.7) 127 (59; 175) (112; 145) n=68 | 0.29 | 6.86 (-5.95; 19.83) |
| Hop for distance absolute per kg (injured side) | 1.71 (0.44) 1.72 (0.5; 2.72) (1.42; 2.02) n=92 | 1.83 (0.48) 1.91 (0.88; 2.72) (1.34; 2.15) n=23 | 1.66 (0.42) 1.67 (0.5; 2.47) (1.43; 1.93) n=69 | 0.11 | 0.165 (-0.040; 0.378) |
| Hop for distance absolute per kg (uninjured side) | 1.81 (0.42) 1.84 (0.63; 2.84) (1.54; 2.16) n=91 | 1.90 (0.47) 1.99 (0.87; 2.84) (1.46; 2.26) n=23 | 1.77 (0.40) 1.82 (0.63; 2.58) (1.55; 2.06) n=68 | 0.22 | 0.126 (-0.072; 0.329) |
| LSI (%) | 94.0 (8.6) 95 (68.6; 111.2) (89.5; 100.7) n=91 | 96.1 (7.3) 93 (80; 111.2) (90.9; 101.2) n=23 | 93.3 (9.0) 95.1 (68.6; 111.1) (88.1; 99.7) n=68 | 0.17 | 2.85 (-1.14; 6.98) |
| **Single-leg side hop** |  |  |  |  |  |
| Side hop absolute (injured leg) | 44.0 (16.2) 43 (13; 79) (31; 57) n=87 | 49.6 (15.7) 52 (13; 79) (39; 60) n=23 | 42.0 (16.1) 42 (14; 74) (30; 55) n=64 | 0.06 | 7.62 (-0.14; 15.53) |
| Side hop absolute (uninjured leg) 12m | 46.5 (15.6) 51 (14; 78) (36; 59) n=87 | 51.1 (14.8) 55 (14; 75) (46; 59) n=23 | 44.9 (15.7) 45 (16; 78) (33.5; 58.5) n=64 | 0.10 | 6.27 (-1.12; 14.00) |
| Side hop absolute per kg (injured side) | 0.618 (0.243) 0.638 (0.131; 1.15) (0.404; 0.811) n=87 | 0.706 (0.241) 0.747 (0.131; 1.15) (0.554; 0.873) n=23 | 0.586 (0.238) 0.584 (0.187; 1.106) (0.386; 0.759) n=64 | 0.046 | 0.120 (0.003; 0.239) |
| Side hop absolute per kg (uninjured leg) | 0.655 (0.237) 0.683 (0.141; 1.2) (0.484; 0.833) n=87 | 0.726 (0.228) 0.779 (0.141; 1.117) (0.554; 0.886) n=23 | 0.629 (0.237) 0.644 (0.213; 1.2) (0.432; 0.806) n=64 | 0.10 | 0.096 (-0.016; 0.213) |
| LSI (%) | 94.2 (13.7) 93.6 (50; 136.8) (88.5; 102.4) n=86 | 97.4 (12.6) 94.8 (75; 136.8) (89.5; 105.3) n=23 | 93.0 (14.0) 93.4 (50; 122.7) (87; 100) n=63 | 0.18 | 4.47 (-2.02; 11.16) |
| For continuous variables, the mean (SD)/median (min; max)/(Q1; Q3)/n= is presented. | | | | | |
